# Acute Postcardioversion Left Atrial Appendage Stasis During Left Atrial Appendage Closure Procedures

**DOI:** 10.1016/j.jaccas.2026.110205

**Published:** 2026-09-11

**Authors:** Alexander Ivanov, Divyanshu Mohananey, Joshua Meskin, Marcie Berger

**Affiliations:** a CaroMont Heart & Vascular, Gastonia, North Carolina, USA; b Department of Cardiology, Medical College of Wisconsin, Milwaukee, Wisconsin, USA

**Keywords:** atrial fibrillation, catheter ablation, spontaneous echo contrast, thrombus, transesophageal echocardiography, ultrasound enhancing agent

## Abstract

**Background:**

Acute postcardioversion left atrial appendage (LAA) low-flow states are recognized but likely underdetected, as repeat appendage imaging is not routine after sinus restoration.

**Case Summary:**

Three patients undergoing left atrial appendage closure (LAAC) developed new LAA spontaneous echo contrast, dense spontaneous echo contrast/sludge, or a persistent filling defect suspicious for thrombus within minutes of cardioversion. Ultrasound enhancing agent distinguished slow complete opacification from a persistent filling defect, guiding individualized deploy-vs-abort decisions.

**Discussion:**

LAAC uniquely permits appendage imaging immediately before and after cardioversion, and serial intraprocedural transesophageal echocardiography offers a rarely available window on acute LAA stasis. Alternative contributors, including ablation, transseptal instrumentation, and anticoagulation strategy, warrant consideration, particularly in combined atrial fibrillation ablation/LAAC workflows. Prospective studies should define prevalence, risk markers, and mitigation strategies.

**Take-Home Message:**

Serial transesophageal echocardiography during LAAC can reveal acute postcardioversion LAA stasis; ultrasound enhancing agent can differentiate slow flow from thrombus and guide individualized deploy-vs-abort decisions.

## Visual Summary

**Figure:**
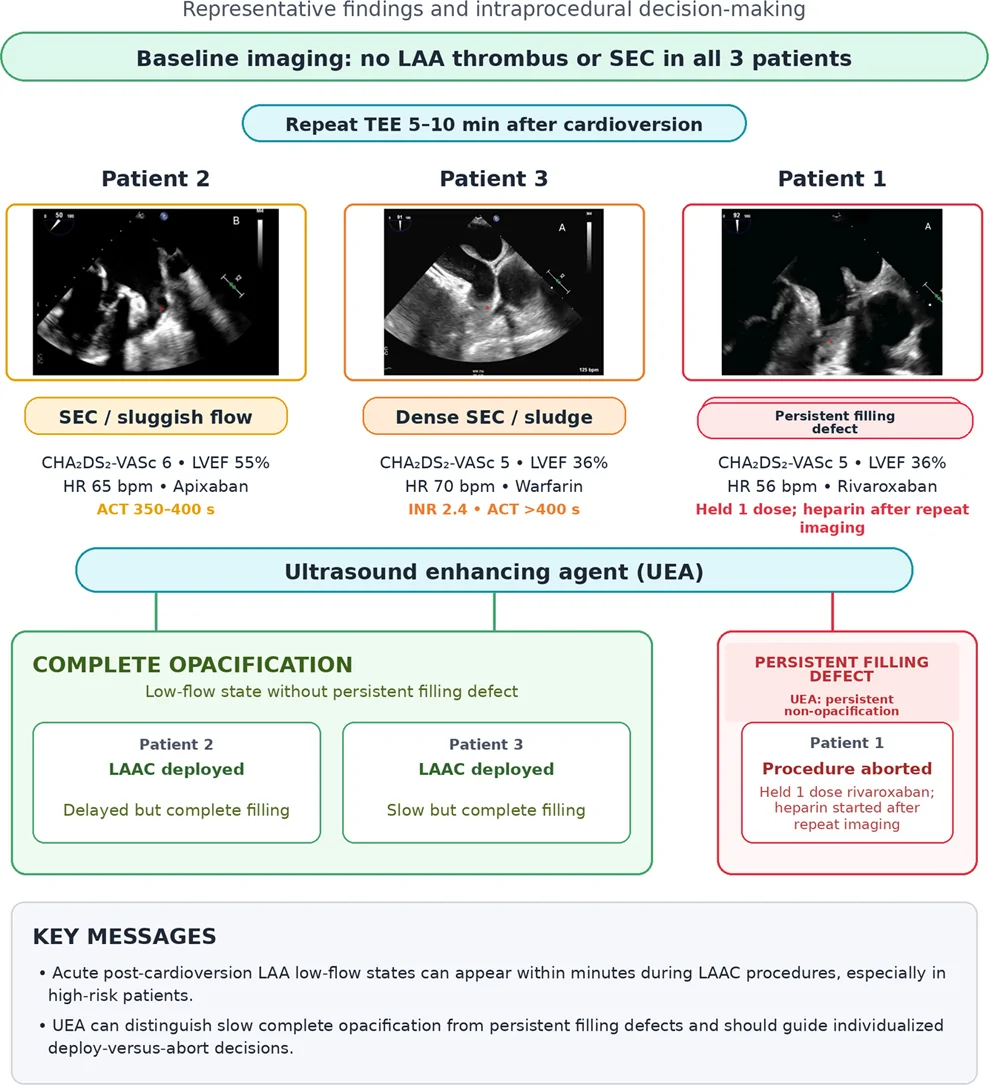

## History of Presentation

All 3 procedures were performed under general anesthesia, and all patients received 324 mg of aspirin preprocedurally. Procedural and imaging equipment is summarized in Table 1.

**Table 1:** Equipment List

Category	Equipment	Manufacturer
TEE	EPIQ CVx system with X8-2t probe	Philips
Ultrasound enhancing agent	Optison (perflutren protein-type A microspheres)	GE HealthCare
Cardioverter/defibrillator	R Series defibrillator	ZOLL Medical
Pulsed field ablation	Farapulse system	Boston Scientific
LAAC device	Watchman FLX PRO device and access sheath	Boston Scientific
Transseptal access	VersaCross Connect Access Solution for FARADRIVE (RF wire and transseptal dilator); RFP-100A RF puncture generator	Boston Scientific
Temporary pacing	Josephson quadripolar catheter	Abbott
Pharmacologic agents	Unfractionated heparin; isoproterenol; aspirin	—

This table summarizes the procedural and imaging equipment used across the 3 cases, including the TEE platform, UEA, cardioverter/defibrillator, ablation system, LAAC device, transseptal access tools, pacing catheter, and pharmacologic agents. The list is intended to support reproducibility of imaging assessment and procedural workflow.

LAAC = left atrial appendage closure; TEE = transesophageal echocardiography; UEA = ultrasound enhancing agent.

### Patient 1

A 77-year-old man with persistent atrial fibrillation (AF), arrhythmia-mediated cardiomyopathy, recurrent falls, and seizure disorder presented for elective standalone left atrial appendage closure (LAAC) (CHA_2_DS_2_-VASc 5, HAS-BLED 4). He had undergone AF ablation 1 year previously and now presented with new atrial tachycardia with 2:1 conduction and a ventricular response rate of 90 beats/min, symptomatic with dizziness. In sinus rhythm, his left ventricular ejection fraction (LVEF) had previously normalized to 57%. He was compliant with rivaroxaban for >4 weeks preprocedure, holding 1 dose preoperatively per institutional protocol.

Preprocedural transesophageal echocardiography (TEE) demonstrated no thrombus or spontaneous echo contrast (SEC) in the left atrial appendage (LAA) (Figure 1A, Video 1); LVEF was 36%, reduced from 57% in previous sinus rhythm, consistent with recurrent tachycardia-mediated cardiomyopathy, and left atrial volume index (LAVI) was 44.85 mL/m^2^. The TEE probe was withdrawn and direct current cardioversion was performed with successful conversion to sinus bradycardia, heart rate 56 beats/min. When the TEE probe was repositioned approximately 5 to 10 minutes later, a new persistent echogenic filling defect suspicious for thrombus was visualized in the LAA (Figure 1B, Video 2). Systemic heparin had not yet been administered at the time of cardioversion because this was a standalone LAAC procedure without preceding transseptal access. Heparin 100 U/kg intravenously (IV) was then administered to achieve an activated clotting time (ACT) >350 seconds, and isoproterenol 4 μg/min IV was initiated for chronotropic and inotropic support, increasing the heart rate to 87 beats/min. The LAA was monitored for 15 minutes without interval change. Ultrasound enhancing agent (UEA) demonstrated a persistent filling defect, suspicious for thrombus (Figure 1B). The defect was considered suspicious for thrombus rather than transient low-flow or sludge based on 4 features: a portion appeared firmly adhered to the LAA apex and remained stationary, without the dynamic, shifting motion of sludge or SEC across the cardiac cycle; it persisted despite reduction of the 2D gain setting; it was consistently present with a similar structural appearance throughout multiplanar imaging; and, following UEA administration, it remained with a sharp, distinct border for more than 20 cardiac cycles. Pre- and postcardioversion images were acquired under comparable conditions, within 4° of the same omniplane angle and with identical 2D gain, depth, frequency, focal zone, time-gain compensation, and harmonic settings. Baseline precardioversion LAA emptying velocity had not been obtained; after the defect was identified, LAA emptying velocity was measured at 18 cm/s. The LAAC procedure was aborted. Rivaroxaban was resumed, and repeat TEE performed 8 weeks later demonstrated no left atrial or LAA thrombus; LAAC was subsequently rescheduled.

**Figure 1:**
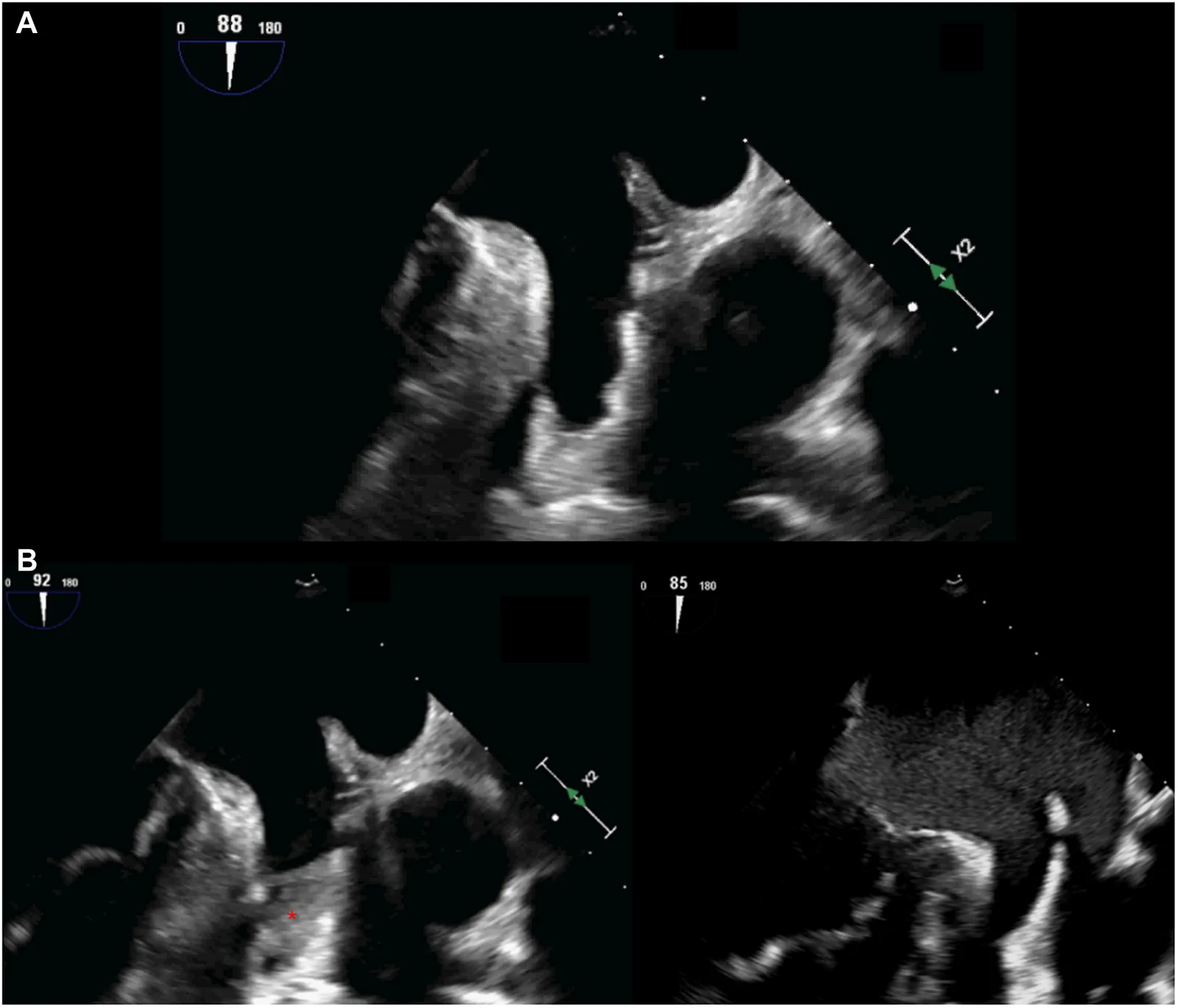
Pre- and Postcardioversion Left Atrial Appendage Imaging (Patient 1) (A) Precardioversion transesophageal echocardiography demonstrated no thrombus or spontaneous echo contrast. (B) Repeat postcardioversion imaging demonstrated a new persistent echogenic filling defect suspicious for thrombus (asterisk); ultrasound enhancing agent demonstrated persistent sharply marginated nonopacification, supporting a persistent filling defect suspicious for thrombus.

### Patient 2

A 78-year-old woman with coronary artery disease, stage 3 chronic kidney disease, mitral annular calcification with mild mitral stenosis, persistent AF, and LVEF 55% presented for elective concomitant AF ablation and LAAC (CHA_2_DS_2_-VASc 6, HAS-BLED 3) because of symptomatic AF and bleeding tendency on oral anticoagulation. She was compliant with apixaban for >4 weeks preprocedure, holding 1 dose preoperatively per institutional protocol. Presenting rhythm was AF with ventricular rate of 59 beats/min.

Preprocedural TEE demonstrated no thrombus or SEC in the LAA (Figure 2A); LAVI was 54 mL/m^2^. The TEE probe was removed and AF ablation was initiated. Heparin 100 U/kg IV was administered with subsequent boluses to maintain ACT 350 to 400 seconds, monitored every 5 minutes until therapeutic and every 10 minutes thereafter. Transseptal puncture was performed; mean left atrial pressure was 19 mm Hg. Pulmonary vein isolation and posterior wall isolation were completed with pulsed field ablation. The patient remained in AF, so direct current cardioversion was performed, restoring sinus rhythm with heart rate 65 beats/min. The ablation transseptal sheath was exchanged for the LAAC delivery sheath, and a pigtail catheter was positioned at the LAA ostium in preparation for device deployment.

**Figure 2:**
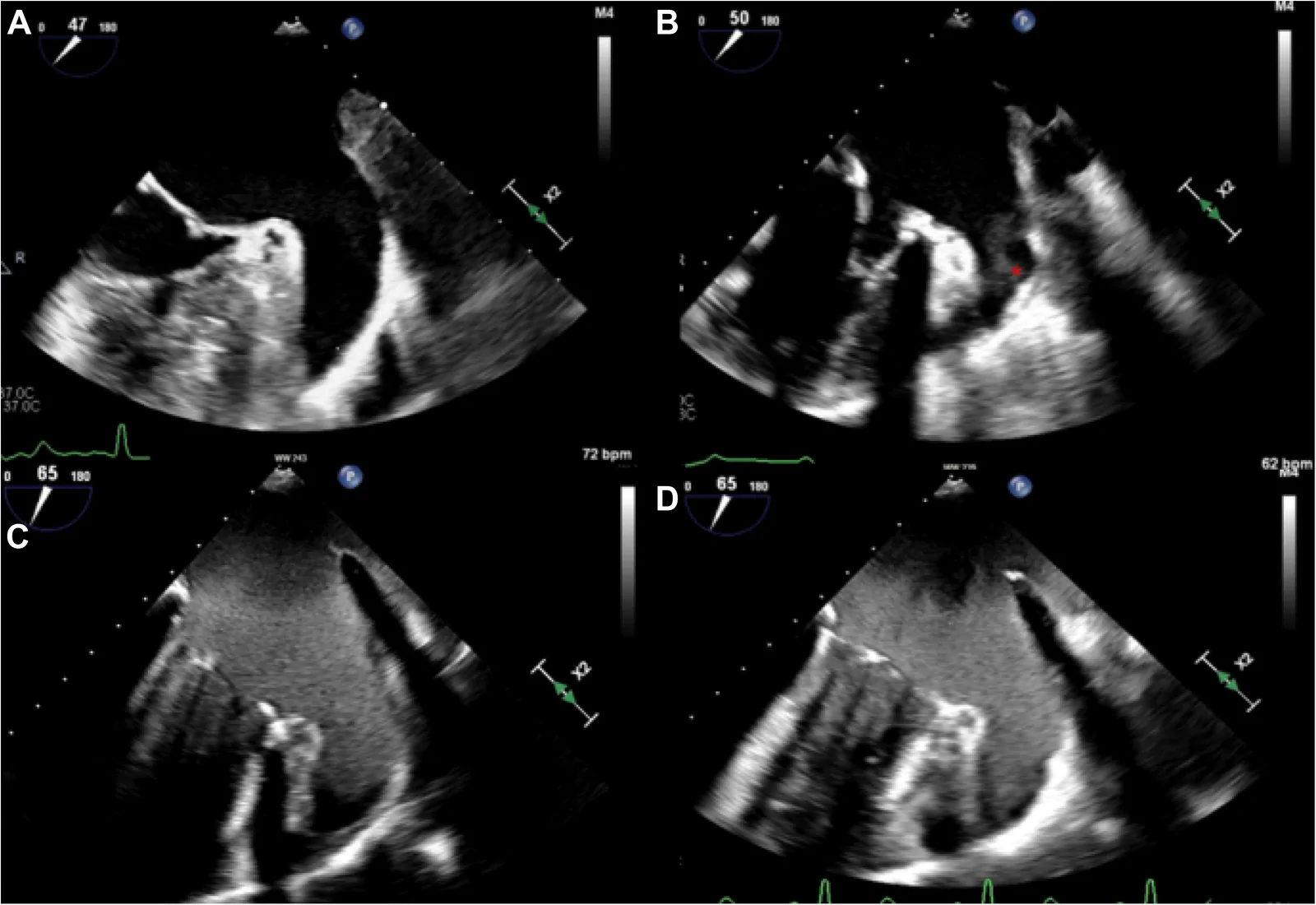
Pre- and Postcardioversion Left Atrial Appendage Imaging (Patient 2) (A) Pre-ablation at omniplane angle 47° demonstrating no thrombus. (B) Postcardioversion at omniplane angle 50° demonstrating new spontaneous echo contrast (asterisk). (C and D) Postcardioversion at omniplane angle 65° in an optimized appendage imaging plane demonstrating delayed but complete filling with ultrasound enhancing agent.

Upon reinsertion of the TEE probe approximately 5 to 10 minutes after cardioversion, new LAA SEC and sluggish flow were noted (Figure 2B). UEA was administered and demonstrated delayed but complete opacification of the LAA (Figures 2C and 2D), consistent with dense SEC/sludge without a persistent filling defect. The LAAC device was subsequently deployed.

### Patient 3

A 76-year-old man with persistent AF, type 2 diabetes mellitus, hypertension, coronary artery disease, ischemic cardiomyopathy (LVEF 36%), and cerebral aneurysm presented for concomitant AF ablation and LAAC (CHA_2_DS_2_-VASc 5). LAVI was 62.2 mL/m^2^. He had previously developed LAA thrombus while on rivaroxaban and was accordingly transitioned to warfarin, with subsequent computed tomography demonstrating resolution of LAA thrombus. After failing amiodarone for sinus rhythm maintenance, and with concern regarding long-term anticoagulation safety given his cerebral aneurysm, he elected to proceed with a concomitant procedure.

He presented for preoperative TEE the day before the procedure with an INR of 3.1. Preoperative TEE confirmed no LAA thrombus. On the day of ablation, INR was 2.4. All weekly INR values were >2.0 for the 4 weeks preceding the procedure. Initial rhythm was AF with ventricular rate of 85 beats/min.

Given therapeutic anticoagulation with warfarin, a reduced initial heparin bolus of 85 U/kg IV was given before transseptal puncture, achieving an initial ACT >400 seconds. ACTs were monitored every 5 minutes until therapeutic and every 10 minutes thereafter, with heparin boluses to maintain ACT 350 to 400 seconds. Pulsed field ablation was performed with pulmonary vein isolation and posterior wall isolation. The patient remained in AF, so direct current cardioversion was performed, restoring sinus rhythm with heart rate 70 beats/min. The ablation transseptal sheath was exchanged for the LAAC delivery sheath, and a pigtail catheter was positioned at the LAA ostium.

Upon reinsertion of the TEE probe approximately 5 to 10 minutes after cardioversion, dense SEC and sludge were noted within the LAA (Figure 3A). The patient was subsequently paced at 120 to 130 beats/min. UEA demonstrated slow but complete opacification of the LAA (Figure 3B), supporting absence of a persistent filling defect, and the LAAC device was subsequently deployed.

**Figure 3:**
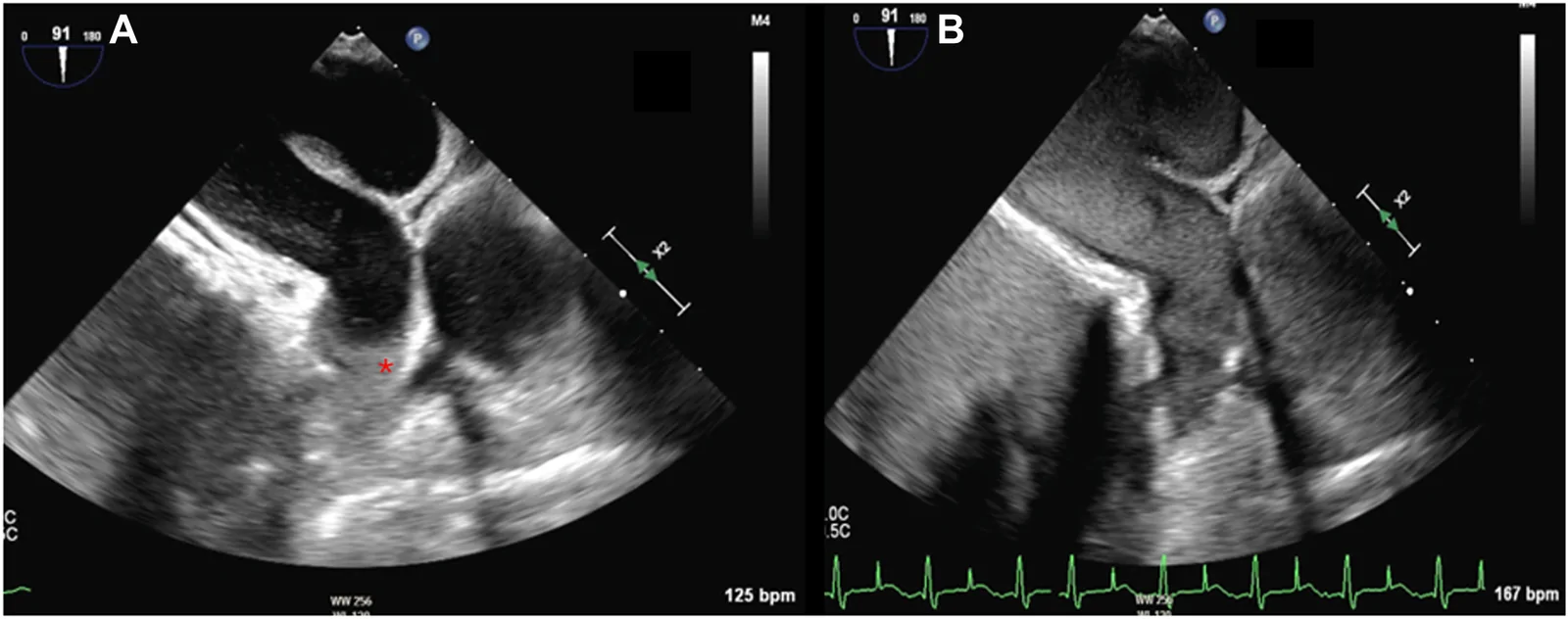
Postcardioversion Dense Spontaneous Echo Contrast and Sludge (Patient 3) (A) Repeat transesophageal echocardiography after cardioversion at omniplane angle 91° demonstrated dense spontaneous echo contrast and sludge within the left atrial appendage (asterisk). (B) Ultrasound enhancing agent showed slow but complete appendage filling during pacing, supporting absence of a persistent filling defect and permitting left atrial appendage closure deployment.

## Discussion

Postcardioversion LAA stasis has been recognized for decades,^1^^,^^2^ but prevalence remains uncertain because repeat TEE is not routinely performed after sinus restoration. The LAAC setting offers a unique opportunity to compare appendage imaging immediately before and after cardioversion, enabling recognition of acute postcardioversion LAA stasis that may otherwise go undetected. Because TEE was already being performed to guide LAAC, repeat imaging within minutes after cardioversion directly visualized acute LAA changes. This continuous intraprocedural imaging window is the principal contribution of the series, offering a rarely available opportunity to observe acute postcardioversion appendage physiology that conventional practice leaves unseen. This setup made it possible to identify low-flow states, distinguish delayed opacification from persistent filling defects with UEA, and incorporate these findings into real-time deploy-vs-abort decision-making. Silent cerebral embolism is a recognized complication of left atrial procedures,^3^ and acute postcardioversion LAA stasis may be one underrecognized contributor.

These observations are best described as acute postcardioversion LAA stasis during LAAC procedures rather than proof of isolated cardioversion-induced thrombogenesis. Cardioversion was the common immediate event, but patients 2 and 3 also underwent transseptal access, left atrial instrumentation, ablation, sheath exchange, and catheter manipulation before repeat imaging. Patient 1 differed in that he held 1 dose of rivaroxaban and had not yet received systemic heparin at the time of cardioversion, whereas patients 2 and 3 had therapeutic ACTs at repeat imaging. Accordingly, the unifying phenomenon in patients 2 and 3 is best regarded as acute intraprocedural LAA stasis arising within a combined ablation–LAAC workflow rather than a cardioversion-specific effect, whereas patient 1—a standalone procedure performed without preceding transseptal access, ablation, or systemic heparin—provides the clearest temporal association with cardioversion. Even so, the appearance of new SEC/sludge in patients 2 and 3 suggests that acute low-flow states can occur despite contemporary periprocedural anticoagulation strategies, including minimally interrupted direct oral anticoagulant and uninterrupted warfarin regimens, in susceptible patients.^4^^,^^5^

The CLOTS-AF score—a validated risk prediction tool for precardioversion LAA thrombus—identifies reduced LVEF (<50%), elevated LAVI (>34 mL/m^2^), renal impairment, reduced tricuspid annular plane systolic excursion, prior stroke, and AF rhythm as independent predictors.^6^ Impaired atrial contractility, reduced LAA emptying velocity, and slower early postcardioversion heart rates are also associated with increased SEC.^1^ All 3 patients had multiple high-risk features, including elevated LAVI (44.85-62.2 mL/m^2^), AF or atrial tachycardia at presentation, and modest postcardioversion heart rates (56-70 beats/min); 2 had LVEF 36%, patient 2 had stage 3 chronic kidney disease, and patient 3 had a previous history of LAA thrombus on direct oral anticoagulant therapy. These features may identify patients in whom repeat postcardioversion LAA imaging is especially informative.

UEA was central to intraprocedural decision-making in all 3 cases. Complete LAA opacification supported a low-flow state without a persistent filling defect in patients 2 and 3, whereas persistent nonopacification in patient 1 supported a persistent filling defect suspicious for thrombus and prompted procedural abortion. We do not infer safety from UEA alone; rather, UEA provided an immediate method to distinguish slow complete filling from a persistent defect and thereby guide individualized deploy-vs-abort decisions at the point of care.

These cases also generate speculative hypotheses for future study. Patients with high-risk features may warrant heightened attention to the interval between cardioversion and LAA assessment, and procedural sequencing may warrant prospective evaluation. In the COMBINATION trial, LAAC-first sequencing during combined procedures was associated with better composite outcomes than ablation-first sequencing.^7^ Whether earlier appendage exclusion reduces embolic risk from acute postcardioversion stasis is unknown. Delayed cardioversion after interval anticoagulation and chronotropic support are other plausible strategies,^8^^,^^9^ but our series does not establish their efficacy. These concepts are speculative and hypothesis-generating only; a small descriptive series reporting intraprocedural imaging without longitudinal clinical outcomes cannot establish their efficacy or safety, and each warrants dedicated prospective evaluation.

This series has several limitations. The small sample size and single-center design limit generalizability. Cases were identified retrospectively, postcardioversion imaging was not protocolized, and LAA emptying velocities were not systematically measured. Definitions of SEC, sludge, and thrombus remain operator dependent across studies. In this series, SEC, sludge, and thrombus were classified using American Society of Echocardiography criteria^10^: SEC as dynamic, swirling reflections arising from erythrocyte rouleaux; sludge as a dynamic, gelatinous, layered echodensity; and thrombus as a well-circumscribed, uniformly echodense intracavitary mass. In patient 3, the baseline transesophageal echocardiogram was performed preoperatively rather than immediately before cardioversion, so identical pre- and postcardioversion imaging planes could not be confirmed. In patients 2 and 3, imaging was obtained for real-time clinical decision-making rather than according to a prospectively standardized protocol; therefore, identical acquisition settings cannot be confirmed in all cases.

## Conclusions

Acute postcardioversion LAA low-flow states, ranging from SEC/sludge to a persistent filling defect suspicious for thrombus, can appear within minutes during LAAC procedures in high-risk patients. These cases highlight a unique intraprocedural opportunity to detect findings that would otherwise go unseen. UEA can help distinguish slow complete opacification from persistent filling defects and thereby guide individualized deploy-vs-abort decisions. Prospective studies are needed to define prevalence, refine risk prediction, and test workflow strategies such as procedural sequencing, delayed cardioversion, and chronotropic support

**Take-Home Messages**

• Acute postcardioversion LAA low-flow states can appear within minutes during LAAC procedures, especially in high-risk patients.
• UEA can distinguish slow complete opacification from persistent filling defects and guide individualized deploy-vs-abort decisions..

**Visual Summary:**
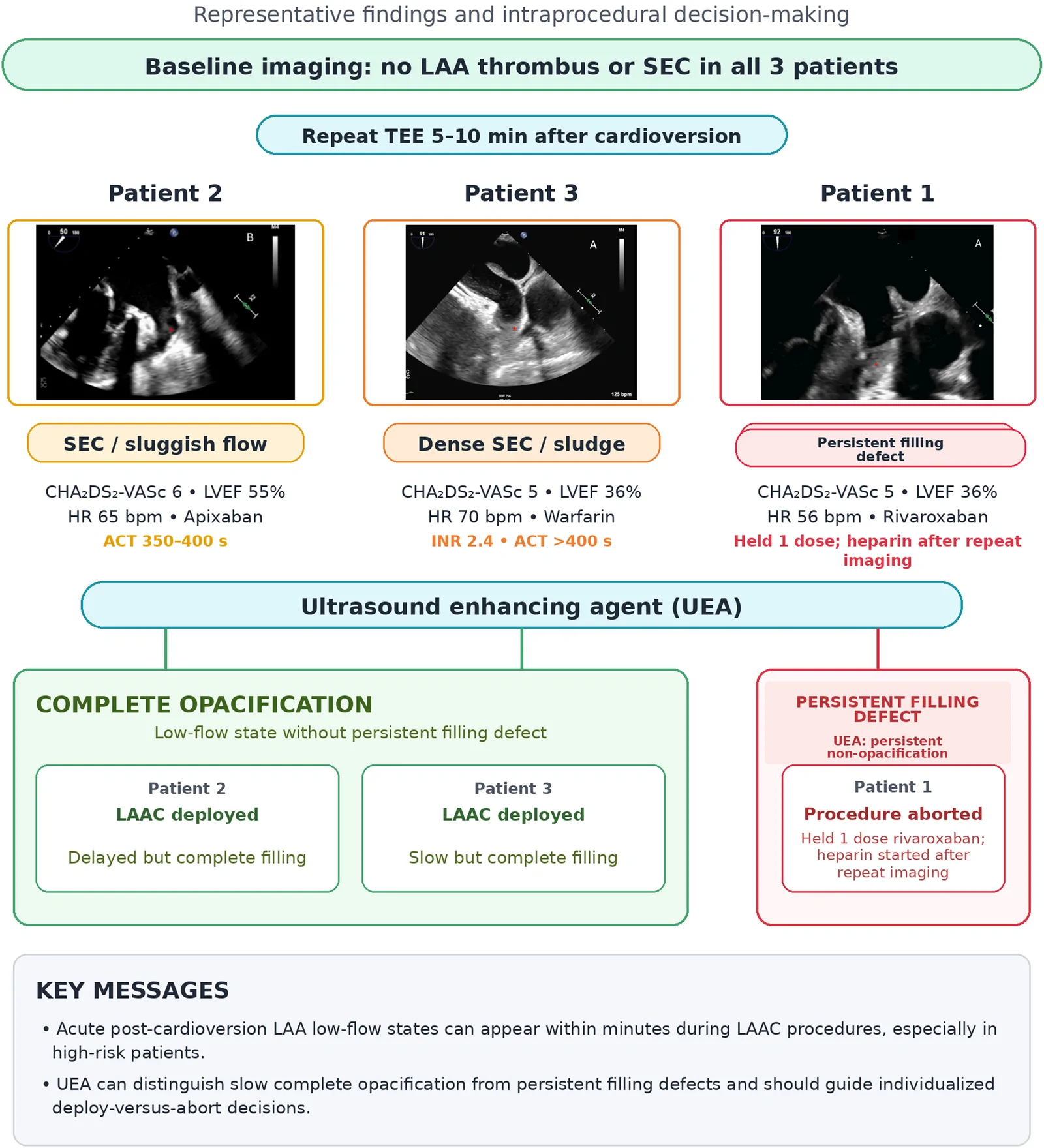
Acute Postcardioversion Left Atrial Appendage Stasis During Left Atrial Appendage Closure Procedures Baseline imaging showed no LAA thrombus or SEC in all 3 patients. Repeat imaging approximately 5 to 10 minutes after cardioversion demonstrated SEC/sluggish flow (patient 2), dense SEC/sludge (patient 3), and a persistent filling defect suspicious for thrombus (patient 1). UEA distinguished slow complete opacification from persistent nonopacification, guiding individualized intraprocedural deploy-versus-abort decisions. ACT = activated clotting time; HR = heart rate; INR = International Normalized Ratio; LAA = left atrial appendage; LAAC = left atrial appendage closure; LVEF = left ventricular ejection fraction; SEC = spontaneous echo contrast; TEE = transesophageal echocardiography; UEA = ultrasound enhancing agent.

## Funding Support and Author Disclosures

The authors have reported that they have no relationships relevant to the contents of this paper to disclose.

## Appendix

Video 1 Precardioversion LAA Imaging (Patient 1) Precardioversion transesophageal echocardiography demonstrates no left atrial appendage thrombus or spontaneous echo contrast. LAA = left atrial appendage; SEC = spontaneous echo contrast; TEE = transesophageal echocardiography.

Video 2 Postcardioversion Persistent LAA Filling Defect (Patient 1) Repeat transesophageal echocardiography approximately 5 to 10 minutes after cardioversion demonstrates a stationary, apically adherent echogenic LAA filling defect suspicious for thrombus. LAA = left atrial appendage; TEE = transesophageal echocardiography.
